# Predictive Value of Imaging Markers at Multiple Sclerosis Disease Onset Based on Gadolinium- and USPIO-Enhanced MRI and Machine Learning

**DOI:** 10.1371/journal.pone.0093024

**Published:** 2014-04-01

**Authors:** Alessandro Crimi, Olivier Commowick, Adil Maarouf, Jean-Christophe Ferré, Elise Bannier, Ayman Tourbah, Isabelle Berry, Jean-Philippe Ranjeva, Gilles Edan, Christian Barillot

**Affiliations:** 1 INRIA-Atlantique, Campus de Beaulieu, Rennes, France; 2 CRMBM-CNRS-Aix-Marseille University, Marseille, France; 3 CHU Rennes, Rennes, France; 4 CHU Reims, Reims, France; 5 CHU Toulouse, Toulouse, France; Cornell University, United States of America

## Abstract

**Objectives:**

A novel characterization of Clinically Isolated Syndrome (CIS) patients according to lesion patterns is proposed. More specifically, patients are classified according to the nature of inflammatory lesions patterns. It is expected that this characterization can infer new prospective figures from the earliest imaging signs of Multiple Sclerosis (MS), since it can provide a classification of different types of lesions across patients.

**Methods:**

The method is based on a two-tiered classification. Initially, the spatio-temporal lesion patterns are classified. The discovered lesion patterns are then used to characterize groups of patients. The patient groups are validated using statistical measures and by correlations at 24-month follow-up with hypointense lesion loads.

**Results:**

The methodology identified 3 statistically significantly different clusters of lesion patterns showing p-values smaller than 0.01. Moreover, these patterns defined at baseline correlated with chronic hypointense lesion volumes by follow-up with an 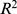 score of 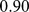.

**Conclusions:**

The proposed methodology is capable of identifying three major different lesion patterns that are heterogeneously present in patients, allowing a patient classification using only two MRI scans. This finding may lead to more accurate prognosis and thus to more suitable treatments at early stage of MS.

## Introduction

Multiple Sclerosis (MS) is an acquired inflammatory, demyelinating disease of the central nervous system. This disease is a major cause of disability among young adults, and is very common in the Northern Hemisphere. Moreover, the high evolution heterogeneity among the patients renders it complex to encompass and to predict at the individual level. Epidemiological and imaging data are showing that MS is a two-phase neurodegenerative inflammatory disease. The early stage is dominated by focal inflammation of the white matter (WM), and the latter stage is dominated by diffuse lesions of WM, gray-matter (GM) and spinal-cord lesions [1], [2]. Large epidemiological studies [1], [3], [4] bring out clinical factors influencing disability during the first phase. These include gender, age at disease onset, number of relapses within the first 2 years and residual deficit after the first relapse. Unfortunately, these factors do not enable prediction of the clinical course at the individual scale. Furthermore, biological and imaging examination still fail to predict accurately disease severity, nature and progression for each patient. To this day, all treatments available are mainly effective only during the early stage, and many arguments suggest that early treatment with disease-modifying drugs can reduce the risk of disability [5]. Because of their significant side effects, some intensive treatments, which are referred to immuno-suppressive drugs, are often used too late [6], [7]. In this context, there is a need for robust and specific markers to characterize the pathology of MS patients and thus identify at the earliest stage those with a high risk of experiencing a more severe disease course in order to tailor the treatment at the individual level [1], [3], [4].

The MS onset can be studied in Clinically Isolated Syndrome (CIS) patients. These patients experienced an initial attack suggestive of demyelination, but do not yet meet the criteria for MS even though they are highly likely to develop it [8]. Macrophage infiltration is an important pattern in inflammatory processes associated with MS. A close link between macrophage infiltration and axonal loss is indeed supported by several studies. There is a spatial link between the presence of active macrophages and axonal injury [9], [10]. Macrophages synthesize free radicals and cytotoxic proteins [11] that are known to cause axonal destruction when they come in contact with brain tissue [12], [13]. This axonal loss may be secondary to mitochondrial injury and subsequent energy failure [14]. Moreover, axonal injury is a major substrate for permanent neurological disability in patients [9], [15]. This can be evaluated by monitoring patients at the onset of the disease using a novel contrast agent, called Ultrasmall Super Paramagnetic Iron Oxide (USPIO) sensitive to macrophages activity, which has been recently used in preliminary studies in human related to brain tumors, stroke, and MS [16]–[19]. Gadolinium (Gd) and USPIO do not highlight the same phenomenon: Gd reveals non-specific blood brain barrier breakdown, whereas USPIO highlights the inflammatory process related to activated macrophages [16]–[19]. Recent studies have suggested a link between USPIO enhancement patterns [18], [19] and the subsequent occurrence of chronic hypointense lesions. Moreover, it has been demonstrated in MS that the T1 hypointense lesions are the site of higher axonal injury [20] and correlate with disability, and to be more highly correlated with disability than T2 lesions [21], [22]. T2 lesions represent variable degrees of demyelination, gliosis, axonal loss, edema and inflammation, while hypointense lesions are more associated with permanent tissue destruction and therefore have a better correlation with disabilities [21]. All these elements suggest that there is a close link between macrophage infiltration, axonal injury and disability.

The use in MS of contrast agents specific of active macrophages offers an opportunity to better understand the occurrence of irreversible disability and could help to predict it. To our knowledge, no previous study has been carried out using USPIO on CIS patients. Moreover the current classification of patients is based either on their total lesion load or disability index without analyzing the specific lesions evolution, the proposed framework aims at providing more specific figures. The overall challenge is to propose a new clinical and data processing paradigm in order to study MS at the disease onset, to provide a better understanding of the early pathogenic mechanisms and to predict the disease evolution at the patient level. In this context, we propose a new framework, based on a two-tiered classification (a the lesion level, followed by a patient level), to find spatio-temporal patterns in CIS patients, using MRI volumes enhanced by USPIO and by Gd and focusing on the longitudinal evolution of lesions. These clusters of patterns are validated by means of statistical analysis, and a correlation with future chronic hypointense lesion load after 21 months follow-up. T2 Total Lesion Load (TLL) by follow-up is also reported for the sake of completeness. This validation is relevant, as these figures correlate with long-term disabilities [21]–[24].

## Materials and Methods

Twenty-five CIS patients (17 women and 8 men), aged 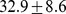 years old at baseline were included between July 2009 and April 2011 in different French centers (Rennes, Marseille, Paris, Toulouse, Reims) based on the following criteria: (i) age between 18 and 45; (ii) occurrence of the first presumed inflammatory demyelinating event in the central nervous system involving either the optic nerve, the spinal cord, a brain hemisphere, or the brainstem; (iii) no previous history of neurological symptoms suggestive of demyelination; (iv) no possible alternative diagnoses (lupus erythematosus, antiphospholipid antibody syndrome, Behcet's disease, sarcoidosis, Lyme disease, cerebral arteritis, brain lymphoma, etc.); (v) patients fulfilling at least the dissemination in space criteria according to McDonald et al [25]; (vi) Expanded Disability Status Scale (EDSS) [26] between 0 and 5 at baseline (vii) first injection of USPIO within three months after the first clinical episode; (viii) no corticoids in the month before USPIO injection and no previous administration of immunomodulatory or immunosuppressive drug; (ix) no previous history of asthma, allergy, or injection of iron oxide particles within the previous 5 months; (x) and no pregnancy.

MR imaging was performed within 3 months after the onset of the disease in different hospitals using either a 3T Verio (Magnetom, Siemens Medical Solutions, Erlangen, Germany) or a 3T Achieva (Philips Medical Systems, Best, The Netherlands) scanner according to a multi-center protocol. The protocol contained axial 2D T1 SE sequences, pre, 5 minutes post Gd injection and 24 hours post USPIO injection, with TR/TE 500/8.4 ms, T2 TSE imaging with TR/TE 6530/84 ms, axial 2D FLAIR with TR/TI/TE 10000/2600/80 ms and 3D FLAIR with TR/TI/TE 5000/1800/273 ms. A 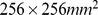 Field Of View was used to cover the whole brain with 44 3-mm 2D slices or a 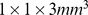 voxel size for 3D acquisitions. The imaging data used in this study was acquired at baseline (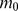), after 3 months (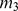), 12 months (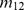) and 24 months (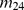). The initial intervals of three months are recommended in order to detect new active lesions according to the temporal dissemination criteria [27], [28]. The latter time points have been used as further validation. Fourteen patients after 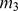 were diagnosed with clinical MS and started undergoing treatments based on interferon beta-1-alpha likely to interfere with the number and extension of lesions, therefore the study limits the observation of the first two time points in order to avoid any drug-related confounding cofactor. Seven patients did not present any active lesions at the first two time points. These patients were excluded from the clustering described in the following sections, but their disease progression in terms of future hypointense lesions and TLLs is also considered, and they are discussed separately in the Discussion section.

Three neuroimage specialists [JCF, IB, AT] reviewed all images independently and blindly from each other. Afterwards, the lesion detections were compared reaching an agreement among the specialists. Subsequently, a fourth physician [AM] performed the manual annotations verifying again the lesions. The chronic hypointense lesions [29] were assessed by analyzing [21] the T1 volumes of 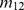 and 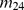, defining a chronic hypointense lesion as an hypointense lesion which is present at 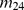 and also visible at 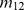. The follow-up TLLs were quantified on T2 volumes at 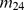. The manual delineations on T2 volumes were also verified on registered FLAIR volumes. The delineations were performed using the MedInria paint tool [30] in a standardized protocol to allow for reproducibility similar to [21]. After one month the procedure was repeated in order to assess intra-observer variability. The following subsections describe the representation of lesions over time and the classification strategy.

### Ethics Statement

The local institutional review board (Comite Consultatif de Protection des Personnes dans la Recherche Biomedicale [CCPPRB], Renns University hospital, France) approved the protocol; and all participants gave their written informed consent.

### Feature space

**Figure 1:**
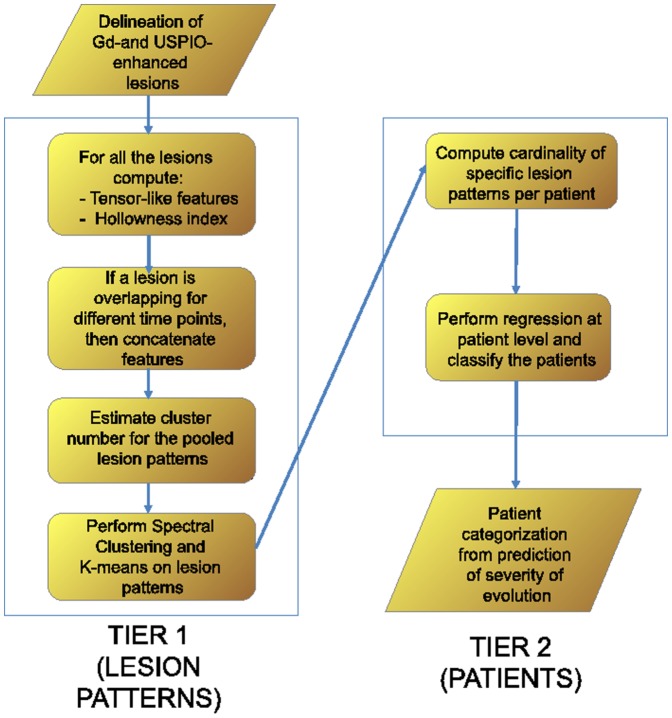
Classification work-flow showing all the steps of the proposed framework.

A specific feature space has been defined for each classification tier: first for the lesion pattern classification, and then for the patient classification. The overall work-flow described in the following sections is depicted in Figure 1.

#### Tier 1: Lesion pattern features

**Figure 2:**
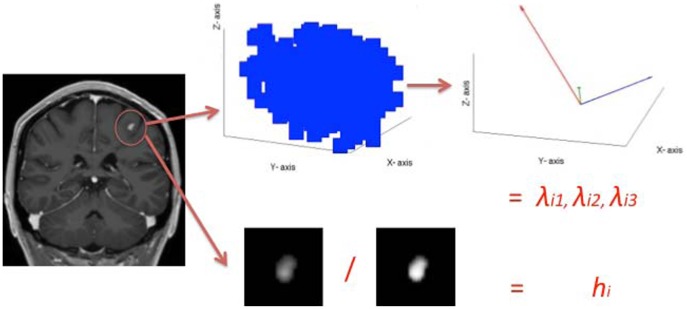
The feature extraction process for a single time point. First all the lesions are delineated, then all the identified voxels are considered to compute the hollow index 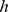 and to build the covariance matrix. Finally, the eigenvalues are obtained from this covariance matrix. The process is repeated for all time points and the lesions which match at the different time point are ordered in the same feature vector.

**Figure 3:**
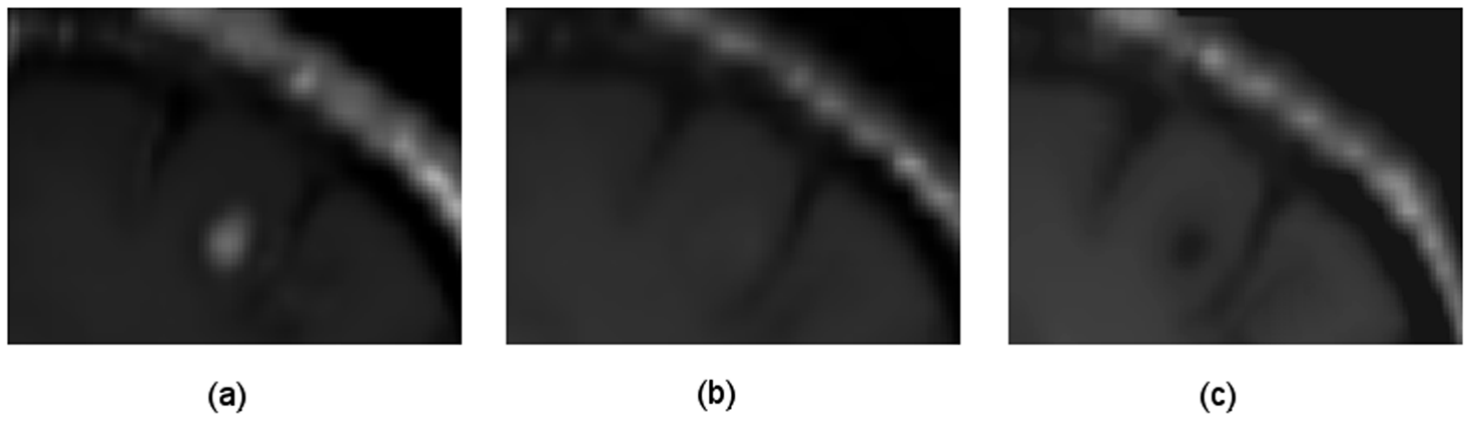
The same lesion at the same time point: (a) Gd-enhanced, (b) USPIO-enhanced and (c) pre-contrast. It can be noticed that the USPIO enhancements are generally very mild compared to the Gd enhancements.

Once the enhanced lesions are manually delineated for all 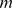 time points (two in our case), they are aligned according to their position at the first time point, using the linear registration tool of MedInria [31]. Several features were deemed to represent the shape evolution of the lesions, such as the volume of each lesion, cell-inspired behavior [32], as well as more advanced shape descriptors such as the Laplace-Beltrami operator [33]. A tensor-like representation combined with a hollowness index 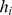 (see equation (1)) appeared to be the optimum choice. Figure 2 depicts the process for a single lesion, and Figure 3 depicts examples of enhanced lesion patterns at different time-points. The ordered eigenvalues of these 3D tensors yield a rotationally invariant feature representation: for each lesion at each time point, the coordinates of the 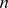 voxels belonging to a specific lesion are collected in the data matrix 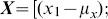 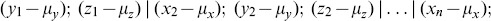 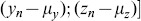 regardless of the intensity of the voxels. Here, 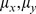 and 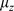 are the mean of the coordinates for the 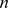 voxels of each lesion, and the size of the matrix 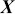 is 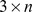. These values are used for defining a covariance matrix 
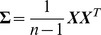
, which can be decomposed in its eigenvalues 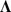 and eigenvectors 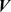: 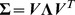. The eigenvectors are neglected since the classification is expected to be orientation invariant, while the eigenvalues can represent the size of the lesions. This representation was also chosen because 75% of the lesions have ellipsoidal shapes or can be approximated by an ellipsoid. The remaining 25% have a ring shape similar to a torus [19], which can also be approximated by a tensor-like representation with same eigenvectors as if the central holes would have been filled but slightly larger eigenvalues. To uniform the representation, the ring-like shape has been considered as filled and the hollowness is measured and concatenated to this information. We refer to the final section for further discussion about this aspect. The hollowness is given by the ratio 
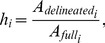
(1)where 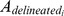 is the lesion area directly obtained by the manual segmentation, and 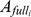 is the same area with filled holes. This ratio should be 1 if there is no hole and becoming smaller than 1 if a hole is present. The experiments were repeated also rounding the hollowness measure to a binary value of either 0 or 1. All three eigenvalues 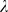 contained in the diagonal of 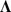 and the hollowness index for all 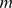 time points are the features representing the temporal evolution of the lesions. This means that for each single lesion, there is a feature vector 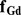 and 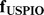 given by 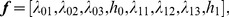
(2)containing the values respectively for the MRI volume Gd-enhanced and USPIO-enhanced, in decreasing order for each time point to be orientation independent, with 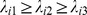 for each of the 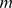 time point. Once the lesions are represented for both contrast agents, the vectors 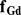 and 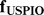 of each lesion are concatenated into a 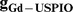 vector. If one lesion has only either 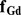 or 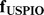, the absence of the features is represented by zeros. The same lesion present in different time points 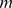, is aligned to the other time representation of itself. This is performed by registering linearly all the manual delineation volumes for the same patient to the volume at the first time point [31]. If there is an overlap between the lesions of the different time point, then they are automatically considered to be the same lesion and collected into the same 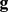 vector. The volumes are also visually inspected to verify correct registration. In a further experiment the tensor-like representation is also compared with a lesion volume representation for each time point, where the feature vector becomes 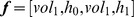 with 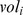 representing the volume of a specific lesion.

#### Tier 2: Patient features

The patient feature vector is given by all the cardinalities of the lesion pattern clusters 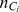 learned during the stage one for each patient 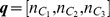, e.g. 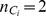 when a lesion pattern is present twice, or 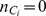 when a lesion pattern does not exist for a given patient. The following subsection describes how the lesion and final patient clusters are obtained.

### Lesion evolutions classification

The feature representations described in the previous subsections are used in a two-tier classification. Initially lesion patterns are identified, then the discovered patterns are used to identify specific patients which could potentially undergo a more severe course. These two steps are performed by an unsupervised clustering algorithm and a regression using the output of the first tier as the input of the second, i.e. the detected lesion patterns 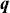 are used as features for a second tier where a regression is performed at the patient level.

K-means, hierarchical clustering and Gaussian Mixture Models (GMM) are well-known unsupervised clustering algorithms [34]. K-means and GMM rely on estimating explicit data models. Hierarchical clustering seeks to build a tree-like hierarchy of clusters based on similarity criteria. These approaches generally work, but they tend to fail when the nature of the data has a complicated structure or the clusters have different sample sizes. One way of coping with these issues, likely to arise in our case, is to use spectral clustering [35]. This latest algorithm is based on the point-to-point similarity matrix rather than on the estimate of an explicit data model. Hence, spectral clustering is adopted as the clustering algorithm in the proposed framework (classification using only the K-means algorithm is also reported).

Yet the choice of cluster number needs to be predefined arbitrarily. Since we are investigating the existence of possible patterns, we have no prior knowledge of the number of clusters and therefore we rely on some validity indices. Many validity indices have been proposed, the reader can refer to [36] for a comparison of some of them. The Dunn index 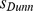 [37] and the Calinski-Harabasz index 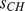 [38] identify sets of clusters that have a small within-cluster variance, and sufficient large between-cluster variance. The Gap-statistics 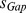 [39] is given by a comparison of the given data-set to an appropriate reference data set drawn from an a priori distribution. The advantage of this validation is that it does not assume the existence of at least 2 clusters, differently from other methods. Another family of methods is strictly related to spectral clustering [40], where the number of clusters 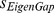 is discovered by analyzing the eigenvalues of the affinity matrix [41] which represents the pairwise distances among all the samples. A robust version of the approach was proposed for the case of noisy or not well separated data [35]. Despite these methods generally work, they do not represent a definitive answer to the challenge of estimating the exact number of clusters and they do not necessarily agree with each other. The choice of the index depends on the nature of the data, on the subsequent used algorithm and on the number of available samples. In absence of a priori knowledge justifying the choice of one measure on the others, it is possible to combine different measures since they can capture certain aspects of a clustering solution [42] in a decision fusion as 
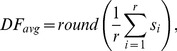
(3)where 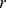 is the number of used scores, and 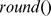 is an operator which approximates a number to the nearest integer. In the reported study, 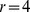 and the values of 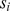 are reported in Table 1. Once the number of clusters is identified, spectral clustering [41] coupled with K-means in the eigenspace is performed. It is worth mentioning that with the self tuning spectral clustering implementation [35], it is possible to have a unique framework comprising validity index, spectral representation and K-means. In the reported experiments, all the mentioned validity indices are computed as well as the decision fusion score (3). The final decision fusion score is considered as the reference score, though the existence of the resulting clusters is additionally validated as described in the Statistical Analysis Section.

**Table 1:** Optimum number of clusters estimated on the tensor-like features using the different validity indices and the rounded mean as described in Section 0 which represents the majority voting.

Feature	n.cluster 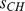	n.cluster 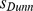	n.cluster 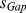	n.cluster 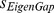	n.cluster 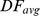
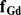	2	2	1	3	**2**
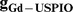	3	2	3	3	**3**

### Patient classification

Once the clustering of the lesions patterns is performed, the detected 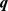 vectors representing the cardinalities of the present lesion patterns per patient can be used to identify specific patients. The correlation between lesion patterns in patients and hypointense lesions is given to relate the patient classification to their disease severity aiming at the prediction of the most severe cases. A regression model [43] was used to examine the correlation between the number of detected lesion patterns and the future chronic hypointense lesion load: 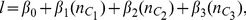
(4)the coefficient 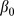 can be neglected because by construction of the model there will be at least one non-zero 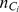 parameter, and 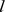 represents the chronic hypointense for a specific patient. The model examines the influence of the detected number of lesion patterns, based on the assumption that some lesion patterns are more indicative of disease severity [44]. The 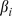 parameters were estimated by minimizing the Least-Squares linear regression given by 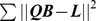, where 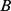 is the vector containing all the 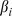, 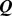 is the matrix containing all the number of patterns per patient 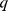 and 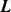 is a vector containing the relative hypointense lesion volume 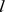.

### Statistical analysis

The statistical significance of the lesion clusters is assessed by computing the cluster separation [45], which is defined as the average distance between each cluster centroid and the other centroids similarly to the Dunn and the Calinski-Harabasz index, and testing this measure against the Null hypothesis that these clusters do not differ, similarly to the Gap-Statistics. More specifically, the cluster separation was computed using the formula reported in [46]

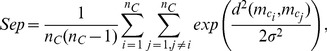
(5)where 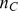 is the number of clusters, 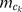 is the centroid of the cluster 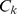, 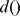 is the distance used by the clustering, and 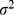 is the between-clusters variation obtained by pooling together the samples of two different clusters examined in turn. The p-values are computed for each cluster considering a rank analysis using 1000000 re-sampled permutations of cluster separation. Considering the different sample sizes of the obtained clusters, the p-values were computed considering both cases of randomization test and permutation test [47]. In the randomization test, all samples are assigned to the clusters with no prior assumption. In the permutation test, the number of samples per cluster is assumed to be unchanged, though there is no evidence that the number of samples within the clusters is correct.

The goodness of fit of a regression model can be assessed through the 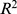 coefficient (also known as Coefficient of determination) [43]. The 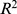 coefficient measures how well the regression line approximates the real data points. An 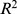 of 1.0 indicates that the regression line perfectly fits the data. The most general definition of the coefficient is 
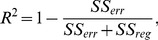
(6)where 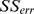 is the sum of squared differences between the predicted 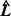 and the reference 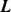 values. 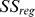 is the sum of squared differences between the predicted values and the mean value of 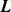. This measurement is repeated in a leave one patient out cross-validation manner, by repeating the computation removing one of the lesions or one of the patient each time. The reported values are the mean and variance of all the obtained 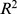 coefficients for both the cases of used features 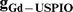 and 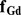 only. The clusters obtained using the 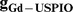 features are also compared to the clusters obtained using 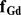 only. This is assessed by computing p-values based on the Z-scores of the samples means.

## Results

In this section the results of the two-tiered classification are reported. First the clinical observations are reported, and then the results for the lesion pattern clusters and the patient groups.

### Clinical results

The mean EDSS score of all twenty-five patients at baseline was 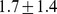. Fourteen patients were assessed RRMS according to the McDonald 2005 criteria [25] already by 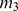, whereas four converted to RRMS by 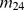. Of the seven patients which did not present either Gd or USPIO active lesions at the two baseline time points, only one was assessed RRMS by 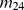 and the remaining six were still CIS by 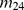.

Comparing the manual annotations of the enhancement for both contrast agents for the same lesions, the USPIO-enhanced lesions show a different behavior than the Gd-lesions and are often visible as a mild ringing around the Gd-lesions as depicted in Figure 3.

The expert radiologist manually delineated 103 Gd-lesions and 24 USPIO-lesions. Only one lesion was enhanced by USPIO and not Gd, such a lesion pattern was grouped in the cluster C1 described in the following section. By examining which of the active lesions converted to hypointense lesions by 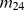, it was noted that generally half of USPIO-enhanced ringing lesions converted to hypointense lesions at 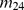 while the other half appeared to recover to isointense, whereas 80% of Gd-enhanced focal lesions converted to hypointense lesions. The lesion volumes are reported per patient in Table 2. The intra-observer variability was assessed by computing an intraclass correlation coefficient of the hypointense lesion load, which gave a value of 0.88.

**Table 2:** List of patients with the relative detected patterns, hypointense lesions volume and TLL in 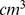 by follow-up (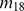), ordered according to chronic hypointense lesions volume.

Patient	lesion clusters (and cardinality)	chronic hypointense at 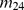	TLL by 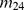	Group
6	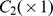 , 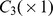 , 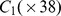	13.40 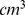	18.9 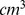	A
24	no active lesions at m0 and m3	3.35 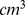	4.63 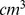	C
11	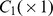	1.80 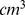	9.70 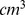	B
4	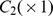 , 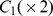	1.77 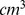	4.29 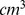	A
16	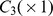	1.46 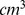	4.70 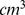	A
9	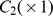	1.42 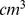	6.72 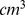	A
10	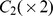 , 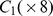	1.37 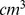	4.82 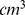	A
25	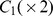	0.96 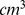	3.32 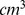	B
7	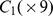	0.82 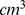	2.10 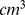	B
18	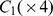	0.75 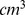	3.40 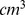	B
21	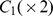	0.74 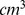	3.50 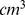	B
17	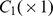	0.52 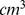	1.18 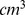	B
2	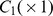	0.46 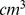	1.90 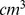	B
12	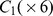	0.34 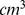	2.10 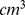	B
8	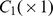	0.31 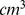	1.14 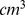	B
3	no active lesions at m0 and m3	0.28 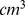	0.98 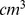	C
19	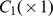	0.27 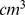	1.73 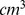	B
20	no active lesions at m0 and m3	0.18 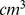	0.54 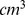	C
5	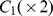	0.14 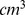	1.70 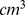	B
15	no active lesions at m0 and m3	0.13 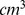	0.68 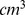	C
13	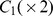	0.12 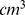	3.54 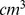	B
1	no active lesions at m0 and m3	0.12 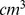	1.18 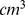	C
22	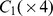	0.10 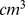	1.27 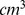	B
23	no active lesions at m0 and m3	0.06 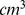	0.49 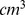	C
14	no active lesions at m0 and m3	0.00 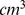	0.29 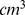	C

The last column pinpoints the different patient groups. Patients of *Group A* present at least one lesion pattern belonging either to the cluster 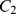 or to the cluster 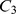, which it is considered an indication of severe condition. Patients of *Group B* do not present any lesion pattern either 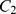 or 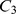. The patients of *Group C* are the patients which do not present active lesions at the first two time points.

### Results lesion pattern classification

The clinical value of USPIO is assessed comparing the results obtained using the 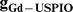 vectors which contain both the Gd and USPIO features and the 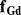 which contain the Gd values only. The comparison to the 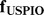 vectors is not feasible because the matrix containing the collection of such vectors will be very sparse. In fact, due to the small number of USPIO lesions compared to Gd lesions, the matrix will be full of zeros and with very few values. Such a matrix is not usable from the algorithms used here for the classification since it is pseudo-singular. A first comparison was performed by computing the validity indices respectively for both representations as reported in Table 1. Although it seems there is no clear agreement about the optimal number of clusters according to the different validity indices, there is generally an increase of cluster number using the 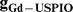 vectors rather than using the 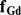 vectors containing only the Gd features. This increase is not related to the increase of dimension - as it can be easily proved using artificial-data - but rather to differences arising from the variable image signatures between the two contrasts agents used jointly. Since the rounded mean of the scores is 3, it is assumed that there are 3 clusters and their presence will be validated as described in the Statistical Analysis section.

**Figure 4:**
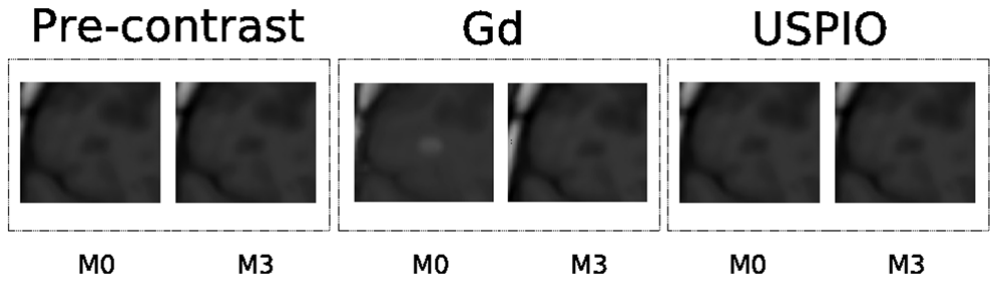
Illustration of a spatio-temporal evolution of the same lesion for both contrast agents and pre-contrast belonging to *C*_1_. In general, 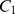 is the less specific which comprises lesions of different dimensions (small, medium, large) appearing at the first time point 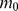 and then disappearing, and generally Gd-enhanced only.

**Figure 5:**
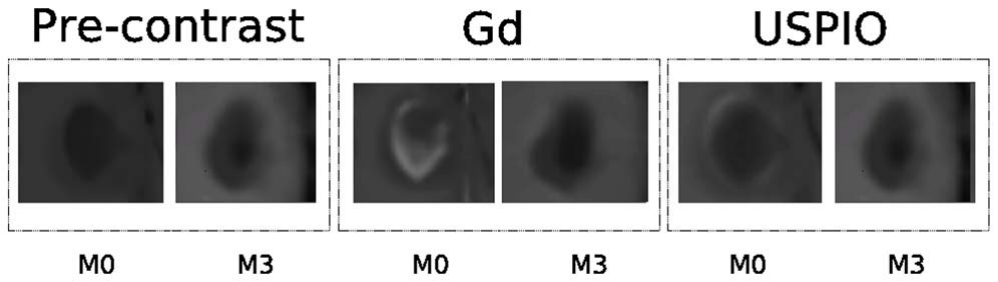
Illustration of a spatio-temporal evolution of the same lesion for both contrast agents and pre-contrast belonging to *C*_2_. In general, 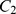 includes relatively medium and large lesions present at both the first two time points, and with co-presence of ringing USPIO and focal Gd enhancement.

**Figure 6:**
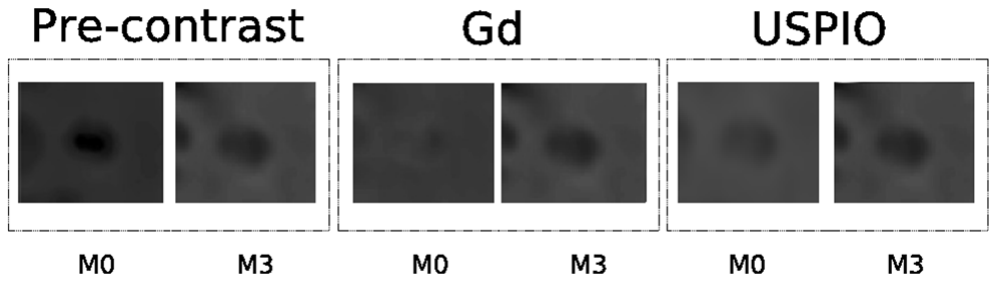
Illustration of a spatio-temporal evolution of the same lesion for both contrast agents and pre-contrast belonging to *C*_3_. In general, 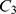 comprises relatively medium lesions present mainly at the first time point with non focal USPIO and Gd enhancement.

The pipeline results highlight whether the same patient has multiple lesions belonging to different lesion clusters 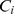. The first identified cluster is the less specific and it comprises different kinds of lesion patterns with similar behaviors while the other two clusters are more specific:

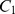
 comprises lesions of different sizes (small, medium, large) appearing at the first time point 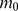 and then disappearing at 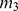, similarly as observed previously [19], and generally only Gd-enhanced (this is the most common pattern observed). Figure 4 depicts a typical example belonging to this cluster.

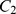
 includes relatively medium and large lesions present at both the first two time points with co-presence of USPIO and Gd, where the Gd-enhanced lesions are focal and the USPIO-enhanced lesions have a ringing behavior. Figure 5 depicts a typical example belonging to this cluster.

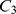
 comprises relatively medium lesions present mainly at the first time point where the USPIO and Gd lesions were both non-focal (ringing or non completely focal) and approximatively of the same size. Figure 6 depicts a typical example belonging to this cluster.

This shows that the clusters differentiate the co-presence of both contrast agents, and not only the size of the lesions.

The statistical significance assessed by the cluster separation [45], for the randomization tests showed for all the clusters a p-value 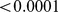. For the permutation tests the p-values were 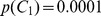, 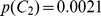, and 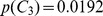.

The experiment was repeated using 1) the Gd features only still using the tensor-like measurements; 2) for both Gd and USPIO features and only Gd using the simpler representation given by the volume of lesions for each time point; 3) using K-means in place of the Spectral clustering algorithm. All these last variations of the experiment produced only two clusters with one cluster comprising relatively large and medium lesions by 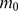 and the other cluster only small lesions. In these variations, the clusters comprising the large and medium lesions were generally larger than the cluster obtained using the main settings (tensor-like representation, both contrast agents and spectral clustering). In fact the lesion patterns comprised by the 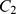 and 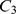 clusters obtained by the main settings were only 7, and the cluster 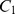 comprised 84 lesion patterns; while with the other settings there were about 20 in the cluster with large and medium lesions and 71 in the remaining cluster.

### Results patient classification

**Figure 7:**
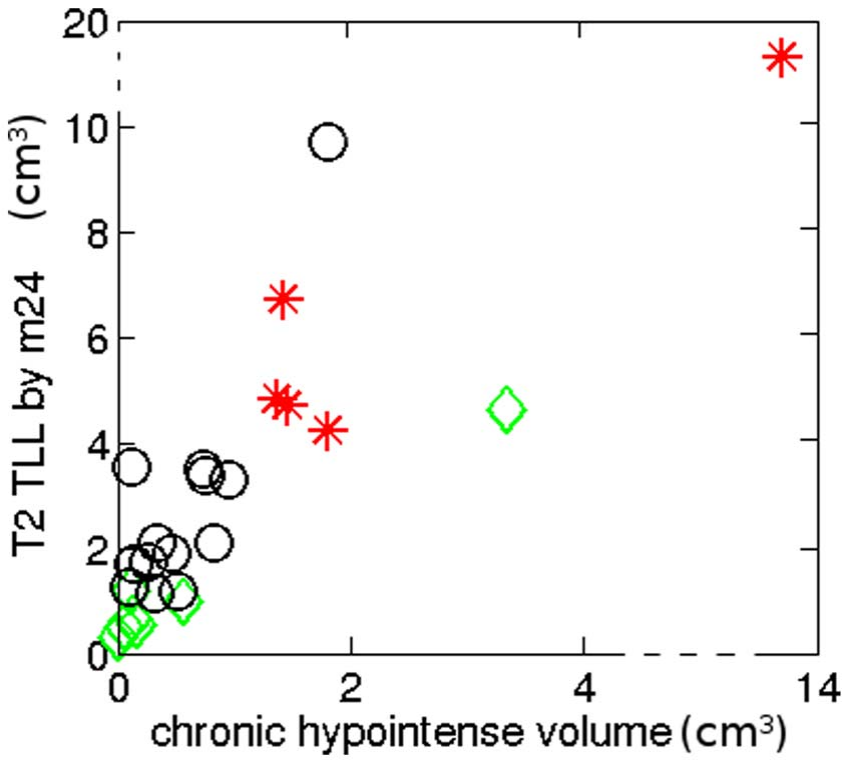
Patients according to their chronic hypointense lesions and TLLs by 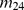. The red stars are patients of *Group A* reported in Table 2 which presented at least one lesion pattern 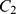 or 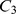, the green diamonds are the patients of *Group C* with no active lesions at the two time points, and the black circles are the reminding patients of *Group B*.

The volume of chronic hypointense lesions - reported in Table 2 - are used to classify patients and to build the regression model of equation (4). Moreover, the table depicts how the lesion patterns correlate with the future volume of the hypointense lesions and TLLs also computed at 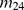, only patients 11 and 24 do not seem to correlate. The classifications have been carried out in a cross-validation manner as well, iteratively removing each time a lesion pattern. The patient groups are also graphically represented according to their chronic hypointense lesion and TLL by 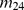 in Figure 7.

Table 3 summarizes the correlation coefficients for the different settings given by the used features and algorithms. The mean 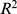 coefficient computed using the equation (6) performed in a cross-validation manner was of 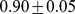. Repeating the entire experiments for 2 clusters using the 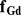 features only (variation 1), the lesion patterns were partitioned into large and medium lesions together at 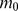 and small lesions at 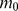 as described in the previous section, and the obtained 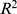 coefficient was 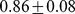. No difference was noticed regarding the use of a continuous measure of the hollow-index and a binary definition as ring-like or filled lesion (namely 0 for ring-like lesions and 1 for filled lesions). The experiments using the volume features for both contrast agent Gd and USPIO and GD only (variation 2) produced the following 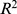 coefficient respectively: 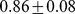 and 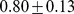. Whereas using only the K-means algorithm (variation 3) yielded 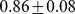 and 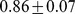.

**Table 3:** 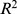
 coefficients for different algorithms and features used.

Settings	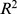 using Gd and USPIO feat.	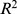 using Gd feat. only
Tensor-like feat. and Spectral clustering	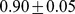	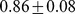
Volume feat. and Spectral Clustering	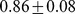	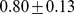
Tensor-like feat. and K-means	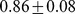	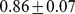
Volume feat. and K-means	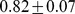	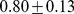

## Discussion

The proposed framework is capable of classifying lesions and MS patients from the very early stages onwards (first 2 MR scans, prior to any drug prescription). This classification correlates closely with the volume of hypointense lesion voxels and to the T2 TLL by 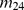. Hypointense lesions represent severe and irreversible tissue destructions which is deemed to correlate to future disabilities [21], [22]. Although less strongly than hypointense lesions, T2 lesions load is considered a predictor figure of future disabilities as well [23], [24]. Moreover USPIO-enhanced lesions show different behaviors than Gd-enhanced lesions. This leads to the hypothesis that USPIO can help to mark lesions with a high risk to decline to a more severe disease course. The proposed machine-learning framework highlighted three main lesion clusters (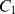, 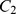 and 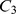). Among them, two were related to a higher TLL and hypointense lesions at 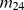. These two components (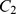 and 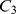) correspond to relatively larger lesions enhanced by USPIO and Gd, in agreement with recent radiological findings showing that lesions enhanced by both contrast agents at baseline were larger and were more likely to persistently enhance at 6-month follow-up compared with those that enhanced only with Gd or USPIO [18].

The 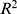 correlation coefficients obtained using both the tensor-like Gd and USPIO features were larger than using the Gd features only. However, studies featuring larger populations are required to fully validate this hypothesis. Nevertheless, we can hypothesize that the disease severity is related to the presence of certain lesion patterns rather than others, in particular to the presence of the lesion patterns 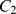 and 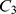, which correspond to very large and active lesions (enhanced by both contrast agents) at the disease onset. By contrast, as expected, patients with only one lesion pattern 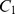 have very few future hypointense lesions and very low TLLs by 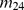. In fact, this characterization suggests as well that having a specific lesion pattern has an incidence on the future evolution of the disease. Since EDSS correlates to lesion load after several years [21], [22], [24].

Moreover, from these observations, it may be hypothesized that not all the lesions - even if enhanced by Gd - contribute in the same way to the worsening of the disease. Disability may not be directly related to a blood brain barrier breakdown, but rather to how the brain overall copes with the damages generated by MS [44]. The presence of specific lesion patterns with co-presence of both Gd and USPIO may be indicative of a more severe course represented by more hypointense lesions and relatively large TLL. The number of active lesions does not necessarily provide such information, therefore a regression model based on the detected clusters - similarly to [48] - was devised.

Contrary to [17]–[19], more Gd-enhanced lesions than USPIO-enhanced lesions were detected. This difference could be motivated by one or more of the following reasons:

- Different cohorts are used. In previous studies, the patients were at a more advanced stage of the disease (RRMS or progressive MS). Moreover, Vellinga et al. [19] included patients with active lesions on MRI and Dousset et al. [17] included patients with active relapse.
- Most of the RRMS patients may be under treatment, which reduces the number of Gd-enhanced lesions.
- The studies have different time interval between MRI scans.

The observation that generally half of USPIO-enhanced ringing lesions converted to hypointense lesions at 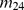 while the other half tends to recover to isointense, whereas 80% of Gd-enhanced focal lesions with no USPIO enhancement converted to hypointense lesions, is partially in line with the results reported in [19], suggesting that some cases - namely USPIO-enhanced ring-like lesions - exhibit a return to isointense lesions. However, the patients presenting at least either one 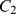 or 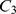 lesion pattern seem to have more hypointense lesions by 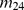, this is probably related again to the fact that these patterns indicate high activity [18]. Beyond the clinical validation, the classification method itself is significantly novel and of general relevance independently from the specific data-set.

The tensor-like representation for lesions is motivated by the rotationally invariant eigenvalues and the analogy to PCA. The detected clusters proved to be statistically significant in our dataset. However, both the tensor-like and volumetric representation can oversimplify the complexity of the lesion enhancement even if still informative. To complement this representation a clear indication of the hollowness of the lesion has been added in such a way to discriminate focal from ringing-like lesions. Future works can comprise to use features obtained using the Laplace-Beltrami operator [33] which could simplify the issue of the hole in the ringing lesions, other MRI quantitative measurements (such as Magnetization Transfer, Diffusion MRI or relaxometry), and evolution in terms of brain atrophy [49]. In summary, the proposed approach can provide an additional classification of the different types of lesions across patients not provided by lesion load measurement only. This new insight could contribute to patient selection for therapeutic trials focusing on preventing or delaying the development of MS and related disabilities.

## Conclusion

A novel paradigm for spatio-temporal analysis of MS lesions at disease onset has been proposed. It has been applied using a novel contrast agent (USPIO) yielding complementary information compared to Gd. We devised a two-layer classification, first identifying lesion patterns then classifying patients in terms of predictive risk of disease progression according to the lesion patterns. Such an approach is very interesting as it allows to relate parameters extracted from patient images to invisible disease parameters such as future course of the disease.

This patient classification performed at the very early clinical stages of MS correlates closely with hypointense lesions and TLLs by 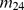, indicating a probable more severe evolution [21], [24]. This finding could lead to a more precise and early prognosis at onset, and therefore to more adapted treatments according to the patient risk profile.
